# Predation risk induces age- and sex-specific morphological plastic responses in the fathead minnow *Pimephales promelas*

**DOI:** 10.1038/s41598-019-51591-1

**Published:** 2019-10-25

**Authors:** Denis Meuthen, Maud C. O. Ferrari, Taylor Lane, Douglas P. Chivers

**Affiliations:** 1 Department of Biology, University of Saskatchewan, 112 Science Place, Saskatoon, SK S7N 5E2 Canada; 2 Institute for Evolutionary Biology and Ecology, University of Bonn, An der Immenburg 1, 53121 Bonn, Germany; 3 Department of Veterinary Biomedical Sciences, WCVM, University of Saskatchewan, 52 Campus Drive, Saskatoon, SK S7N 5B4 Canada; 4 Toxicology Centre, University of Saskatchewan, 44 Campus Drive, Saskatoon, SK S7N 5B3 Canada

**Keywords:** Evolution, Ecology, Evolutionary ecology, Freshwater ecology

## Abstract

Although comprehending the significance of phenotypic plasticity for evolution is of major interest in biology, the pre-requirement for that, the understanding of variance in plasticity, is still in its infancy. Most researchers assess plastic traits at single developmental stages and pool results between sexes. Here, we study variation among sexes and developmental stages in inducible morphological defences, a well-known instance of plasticity. We raised fathead minnows, *Pimephales promelas*, under different levels of background predation risk (conspecific alarm cues or distilled water) in a split-clutch design and studied morphology in both juveniles and adults. In accordance with the theory that plasticity varies across ontogeny and sexes, geometric morphometry analyses revealed significant shape differences between treatments that varied across developmental stages and sexes. Alarm cue-exposed juveniles and adult males developed deeper heads, deeper bodies, longer dorsal fin bases, shorter caudal peduncles and shorter caudal fins. Adult alarm cue-exposed males additionally developed a larger relative eye size. These responses represent putative adaptive plasticity as they are linked to reduced predation risk. Perhaps most surprisingly, we found no evidence for inducible morphological defences in females. Understanding whether similar variation occurs in other taxa and their environments is crucial for modelling evolution.

## Introduction

Traits are highly variable due to phenotypic plasticity, the ability of genotypes to express different phenotypes dependent on the environment^1^. Understanding the patterns of variation in plastic traits is a pre-requirement for comprehending the involvement of plasticity in the evolutionary process^2^, a topic of major current interest^3^. Plastic trait expression can vary due to developmental constraints^4–7^ and differences in selection pressures manifesting across ontogeny^7^ and between sexes^8–12^, but this variation has been largely neglected in empirical research, leading to a distorted view of the impact of phenotypic plasticity on evolution^2^. Well-known examples of phenotypic plasticity are inducible defences^13,14^. These predator-induced morphological defences in prey organisms such as the helmets and defensive spines in predator-exposed *Daphnia*^15–17^ and the body depth response of predator-exposed crucian carp *Carassius carassius*^18^ increase fitness through reduced levels of predation^19,20^. The impact of such inducible defences on the evolution of predator-prey systems is also of current key interest^21^. Predation risk is often most pronounced for certain age and/or size classes^22^ and differs between sexes^23–27^, which together with developmental constraints^4–6^ is theoretically predicted to generate ontogeny-specific and sex-specific morphological antipredator plasticity^28–30^. Nevertheless, most reports on inducible defences largely focus on the expression of defences at a single developmental stage and assess morphology independently of sex, leaving us with a rather incomplete understanding of variation in inducible defences^31–33^. Indeed, recent research suggests that inducible defences are not uniformly expressed but instead display variation among developmental stages and sexes^30^. However, patterns of predator-induced morphological variation may differ between species similar to predator-induced behavioural traits^34^. Thus, using previous studies on inducible defences as a reference without further verification across taxa is likely to misinterpret the degree of plasticity present in nature, which distorts our view of the ability of organisms to cope with fluctuating levels of predation risk^2^.

Here, we aim to study the ontogeny-specificity and sex-specificity of inducible defences in a classical model system that is phylogenetically similar to the crucian carp, the fathead minnow *Pimephales promelas*^35^, a small-bodied cyprinid common to lakes and rivers across North America^36^. Fathead minnows are common prey fish and well-studied in regard to behavioural predator-related adaptations^37–44^ but hitherto no study has addressed inducible morphological defences of fathead minnows. As Meuthen *et al*.^30^, following the theory of Fischer *et al*.^7^, suggest that morphological changes are present only during early development and at the onset of sexual maturation, we focus on studying these two developmental stages in particular. For this purpose, we manipulated perceived predation risk by regularly exposing fish from hatchlings onwards to conspecific alarm cues. Alarm cues are prey-borne cues that are released by injured conspecifics following mechanical damage of the skin, as would typically occur during a predation event^45^. These chemical cues readily disperse across the water column^46^ and represent a reliable indicator of risk that is innately recognized by conspecifics^47,48^. Alarm cues are one of the major factors inducing behavioural^49^ and morphological changes^30,50–52^ that are typical for high-predation habitats. In the present study, we applied a split-clutch design and raised fathead minnows exposed either to chemical cues indicating high-risk conditions (conspecific alarm cues), or control cues (distilled water) five days a week. We then photographed fish at 18 and 180 days age to obtain morphometric information on juveniles and adult fish of both sexes. In fish, common morphological defences are deeper bodies^18,53–55^ that increase prey handling times for predators and thereby increase the probability of a successful escape^18,56^. Another effective induced defence are plastically induced longer and deeper caudal peduncles^30^ that enhance escape locomotion which prevents capture by predators^57,58^. Moreover, the expression of inducible defences varies among ages and sexes^28–30^. Accordingly, we expected first to observe inducible defences that match the previously observed morphological changes but differ between juvenile fathead minnows and those at the onset of sexual maturity. Second, we expected to observe that these defences differ between sexes at the point of maturation.

## Methods

### Ethical note

Experiments complied with Canadian laws, including the Canadian Council on Animal Care (CCAC) guidelines for humane animal use, and were approved by University of Saskatchewan’s Animal Research Ethics Board (Animal Use Protocol: 20170089).

### Experimental fish

The fathead minnow is a small-bodied fish with up to 60–74 mm adult body size and a maximum life expectancy of 2–3 years. As juveniles, minnows form free-swimming shoals whereas as breeding adults, they become fractional spawners with allopaternal care^36^. Fathead minnows become sexually mature after 4–5 months under chronic 26–32 °C temperature conditions^59^ but at a 20 °C average temperature, morphological changes indicating sexual maturity^36^ occur in the first individuals at 6 months age (D. Meuthen, personal observation). Fathead minnows are a common prey fish that frequently co-occur with various predator species^39,43^. Hence, they have evolved various forms of behavioural antipredator plasticity that allow them to respond appropriately to predation risk, making them one of the foremost model systems on antipredator responses^37–44,49,60^. In September 2017, we produced fish for our experiments by rearing *P. promelas* from hatching onwards in a split-clutch design where offspring were subject to different treatments five days a week while all other conditions remained the same: either alarm cues (AC) or a distilled water control (DW). See the electronic supplementary material, §1 for more details on rearing conditions and alarm cue production.

### Photographic documentation

Fish were photographed first after they completed their larval development, at 18 days age, and a second time at the onset of maturity that occurred at 180 days age; fish in different treatments did not differ in their time of maturation (D. Meuthen, personal observation). In total, two replicates of each 10 fish per treatment, originating from 10 different families, were raised. We analyzed 200 photographs (100 per treatment) of juveniles. For adults, we analyzed 267 photographs of fish whose sexes were clearly recognizable (alarm-cue exposed males n = 85, control males n = 72, alarm cue-exposed females n = 53, control females n = 57). A detailed description of the set-up for taking photographs and the process of selection of suitable photographs for analysis is presented in the electronic supplementary material, §2.

### Data analysis

**Figure 1:**
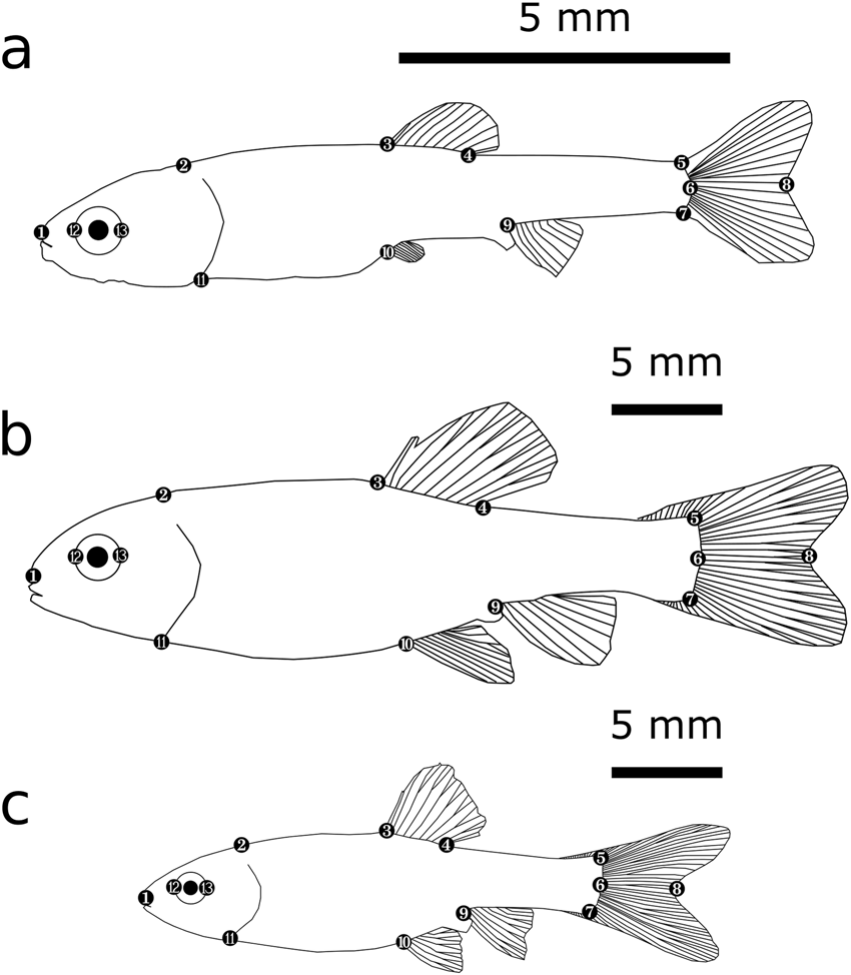
The 13 landmarks used for morphometric analyses in juvenile (**a**), adult male (**b**), and adult female (**c**) *Pimephales promelas*. Traditional morphometric equivalents are: standard length (1‒6), total length (1‒8), body depth (3‒10), dorsal fin base width (3‒4), caudal fin base height (5‒7), eye diameter (12‒13), head height (2‒11), and caudal peduncle length (4‒5). Size standards are displayed in the upper right of each figure.

We placed thirteen landmarks on every photograph (Fig. 1) and analyzed them with geometric morphometrics software so as to extract the canonical variates, principal components and centroid sizes that describe body shape and size as outlined in the electronic supplementary material, §3. This data was then further analyzed in R 3.3.1 (R Core Team, 2018). To reveal treatment- and sex-related changes in body shape, we applied linear-mixed effect models (LMEs; function lme from the R library “nlme”). For juveniles, we used the respective canonical variate, the respective principal component or the respective centroid size as dependent variable, “treatment” (alarm cue-exposed or control) as explanatory variable and “family identity” as random factor to control for genetic variation as made possible by our split-clutch rearing design. For adult fish, we additionally included sex (male or female) as an additional explanatory variable and investigated the relationship between all variables and treatment dependent on sex (“sex” × “treatment” interactions). When at least tendential interactions were present, we split the dataset into two datasets containing one sex each and analyzed treatment effects separately; hence in these analyses, “treatment” was the only explanatory variable. As we used fish from multiple replicates of the same family/treatment combination for adult fish, we needed to control for both genetic and tank-by-tank variation in morphology by nesting “family identity” in “replicate” as random factors throughout analyses concerning adult fish. For all analyses, we visually inspected quantile-quantile plots of model residuals so as to confirm that they did not deviate from normality. All tests of statistical significance were based on likelihood ratio tests (LRT), assessing whether the removal of a variable leads to a significant decrease in model fit according to the Aikake information criterion. P values refer to the increase in deviance when the respective variable was removed.

## Results

### Juvenile fish

**Figure 2:**
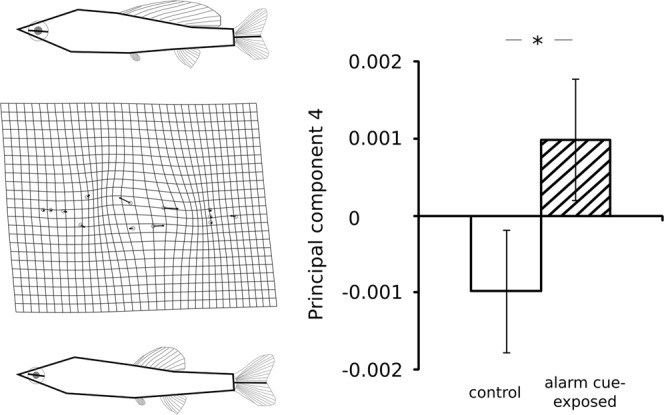
Morphological differentiation along the fourth principal component (PC4, explaining 8.339% of phenotypic variation) of 18-day-old juvenile *Pimephales promelas* subject to different levels of perceived predation risk: distilled water (n = 100; white bar) and conspecific alarm cues (n = 100; dashed bar). Mean values ± SE as well as depictions of the deformation in body shape along the axis are shown. Schematic fins and eyes (gray) are included in these figures to better visualize measured changes in fin position and fin base width. The asterisk indicates p < 0.05.

The first CV axis separates juvenile fathead minnows from the two different treatment groups (LRT, χ² = 21.637, Δdf = 1, p < 0.001, Fig. S1). Shape variation in the first CV axis loaded mainly on a composition of body depth (relative vertical position of dorsal fin landmarks 3,4, anal fin landmark 9, pelvic fin landmark 10 vs. caudal peduncle landmarks 5,6,7 and head landmarks 2,10, see Fig. S1). Comparisons of four principal components revealed no significant differences in the 1^st^ (28.623% of variation, LRT, χ² = 0.004, Δdf = 1, p = 0.952), 2^nd^ (14.340% of variation, LRT, χ² = 0.108, Δdf = 1, p = 0.742) or 3^rd^ principal component (11.205% of variation, LRT, χ² = 0.431, Δdf = 1, p = 0.512). However, we found a significant difference between treatment groups in the 4^th^ principal component (8.339% of variation, LRT, χ² = 5.111, Δdf = 1, p = 0.024, Fig. 2), which largely mirrors the shape deformation present in the first CV axis, thereby confirming a body depth response in alarm cue-exposed individuals (see Figs S1 and 2). All seven following principal components that explained more than 2% phenotypic variance each did not differ significantly between treatments (PC5: 6.674% variation, LRT, χ² = 0.412, Δdf = 1, p = 0.521; PC6: 5.591% variation, LRT, χ² = 0.043, Δdf = 1, p = 0.835; PC7: 5.193% variation, LRT, χ² = 3.442, Δdf = 1, p = 0.064; PC8: 4.463% variation, LRT, χ² = 1.753, Δdf = 1, p = 0.186; PC9: 4.103% variation, LRT, χ² = 0.491, Δdf = 1, p = 0.484; PC10: 2.175% variation, LRT, χ² = 0.424, Δdf = 1, p = 0.515; PC11: 2.023% variation, LRT, χ² = 2.035, Δdf = 1, p = 0.154). Likewise, centroid size did not differ between treatments (LRT, χ² = 1.870, Δdf = 1, p = 0.172).

### Adult fish

**Figure 3:**
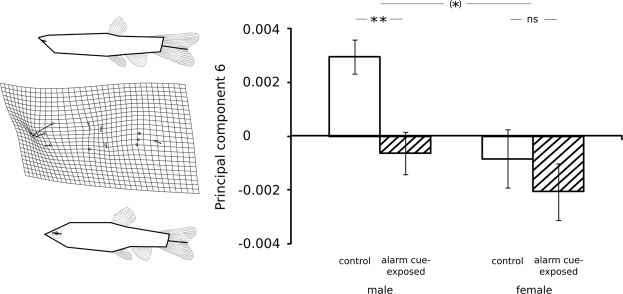
Morphological differentiation along the sixth principal component (PC6, explaining 6.691% of phenotypic variation) of male and female 180-day-old adult *Pimephales promelas* subject to different levels of perceived predation risk: distilled water (male n = 72, female n = 57, white bars) and conspecific alarm cues (male n = 86, female n = 53, dashed bars). Mean values ± SE as well as depictions of the deformation in body shape along the axis are shown. Schematic fins and eyes (gray) are included in these figures to better visualize measured changes in fin position and fin base width. The topmost line above bars represents the interaction between sexes; asterisks denote statistically relevant differences **p < 0.01, *p < 0.1, ns p > 0.1.

Sexes and developmental environments in adult fathead minnows could be separated along the first two CV axes. The first axis, where no sex-by-treatment interaction is present (interaction “sex” x “treatment”, LRT, χ² = 0.445, Δdf = 1, p = 0.505), separates male minnows from female minnows (effect of sex, LRT, χ² = 126.115, Δdf = 1, p < 0.001) while treatment has no significant impact here (effect of treatment, LRT, χ² = 1.071, Δdf = 1, p = 0.301). In the second axis, sexes responded significantly differently to the developmental environments (interaction “sex” x “treatment”, LRT, χ² = 3.944, Δdf = 1, p = 0.047). Male fish from the two treatments were clearly separated by the second axis (LRT, χ² = 23.618, Δdf = 1, p < 0.001) whereas females were not (LRT, χ² = 2.392, Δdf = 1, p = 0.122). In the third axis, we likewise observed a sex-specific response to the treatments (interaction “sex” x “treatment”, LRT, χ² = 14.989, Δdf = 1, p = 0.001). The third axis separated females from the two treatments (LRT, χ² = 7.465, Δdf = 1, p = 0.006), which was not the case for males (LRT, χ² = 2.063, Δdf = 1, p = 0.151). Shape variation in the first CV axis loaded mainly on a composition of belly size (relative position of pelvic fin landmark 10 vs. head landmark 2, dorsal fin landmark 4 and anal fin landmark 9) and caudal peduncle size (relative position of caudal fin landmarks 5, 6, 7). Shape variation in the second CV axis instead loaded mainly on a composition of body depth (relative positions of pelvic and anal fin landmarks 9, 10 vs. dorsal fin landmarks 3,4), head size (relative position of head landmark 2 vs. head landmark 11), eye size (relative position of eye landmark 12 vs. eye landmark 13) and caudal fin fork length (relative position of caudal fin fork landmark 8 vs. caudal peduncle landmarks 5, 6, 7, see Fig. S2). Shape variation in the third CV axis loaded primarily on body depth (relative positions of pelvic and anal fin landmarks 9, 10 vs. dorsal fin landmarks 3,4) and fish posture (relative position of caudal peduncle landmarks 5, 6, 7 to caudal fin landmark 8, see Fig. S3). Comparisons of all eleven principal components that each explained more than 2% of phenotypic variation revealed tendential sex × treatment interactions in principal components 6 and 11 (Table 1). The differences in morphology between sexes explained a large amount of phenotypic variation; significant sex effects were present in principal components 4, 5, 6, 7, 8, 9, 10, together explaining 36.729% of phenotypic variation (Table 1). Instead, significant general treatment effects were present only in principal component 6. Separating data from males and females revealed significant effects of treatment only for males in principal component 6 (Fig. 3, Table 1) that mirror the body depth response of alarm cue-exposed males in the second CV axis (see Figs S2 and 3). No significant treatment effects were observed in either sex during analysis of principal component 11 (Table 1), indicating that the observed female-specific separation along the third CV axis (Fig. S3) is based on noise instead of a treatment effect^61^. Analysis of centroid sizes suggested no sex × treatment interactions or treatment effects but revealed a significant difference between males and females, with males being larger than females (Table 1).

**Table 1:** Results of linear-mixed effect models (LME) analyzing the effect of the developmental environment on the eleven principal components cumulatively describing 90.87% of variation in body shape and centroid size in male and female 180-day-old adult *Pimephales promelas* subject to two treatments that lead to different levels of perceived predation risk. In all analyses, random factors were “family identity” (to account for our split-clutch design) nested in “replicate” (to account for tank effects). The reported p values of the model refer to the increase in deviance upon removal of the respective variable. Asterisks denote significant differences: *p < 0.05, **p < 0.01, ***p < 0.001.

	Δdf	χ²	P
**PC1** (24.231% of variation)			
Sex × Treatment	1	2.387	0.122
Sex	1	3.074	0.080
Treatment	1	1.094	0.296
**PC2** (16.981% of variation)			
Sex × Treatment	1	0.954	0.329
Sex	1	0.377	0.539
Treatment	1	0.568	0.451
**PC3** (10.877% of variation)			
Sex × Treatment	1	0.132	0.716
Sex	1	0.762	0.383
Treatment	1	0.843	0.359
**PC4** (8.448% of variation)			
Sex × Treatment	1	0.775	0.379
Sex	1	20.884	<0.001***
Treatment	1	1.697	0.193
**PC5** (6.854% of variation)			
Sex × Treatment	1	2.189	0.139
Sex	1	8.251	0.004**
Treatment	1	0.330	0.948
**PC6** (6.691% of variation)			
Sex × Treatment	1	3.580	0.059
Sex	1	8.255	0.004**
Treatment	1	6.076	0.014*
*Male fish*			
Treatment	1	10.853	0.001**
*Female fish*			
Treatment	1	0.112	0.738
**PC7** (5.340% of variation)			
Sex × Treatment	1	0.105	0.746
Sex	1	11.210	<0.001***
Treatment	1	0.426	0.514
**PC8** (3.785% of variation)			
Sex × Treatment	1	0.575	0.448
Sex	1	6.423	0.011*
Treatment	1	0.002	0.968
**PC9** (3.113% of variation)			
Sex × Treatment	1	0.269	0.604
Sex	1	7.604	0.006**
Treatment	1	2.328	0.127
**PC10** (2.497% of variation)			
Sex × Treatment	1	1.010	0.315
Sex	1	20.846	<0.001**
Treatment	1	2.041	0.153
**PC11** (2.051% of variation)			
Sex × Treatment	1	3.461	0.063
Sex	1	1.203	0.273
Treatment	1	1.172	0.279
*Male fish*			
Treatment	1	0.100	0.752
*Female fish*			
Treatment	1	1.933	0.164
**Centroid size**			
Sex × Treatment	1	0.393	0.531
Sex	1	21.391	<0.001**
Treatment	1	0.289	0.591

## Discussion

Alarm-cue induced morphological antipredator plasticity was present at both developmental stages but sex-specific plasticity was seen only at the onset of sexual maturity. Plastic morphological responses to the alarm cue-exposure treatment were shown by both juvenile and male but not female *Pimephales promelas*. In juvenile fish, these morphological responses manifested mainly in body depth, dorsal fin base width and caudal peduncle length with alarm cue-exposed individuals developing relatively deeper heads and bodies, longer dorsal fin bases and shorter caudal peduncles as well as shorter caudal fins. Adult alarm cue-exposed males displayed similar responses but additionally developed larger relative eye sizes than controls, a response not observed in juveniles.

The observed plastic development of a deeper head and a deeper body in juvenile and adult male *P. promelas* match the observed plastic responses of another cyprinid, the crucian carp *Carassius carassius*, to predatory habitats^18^, to predator odours^62^ and to conspecific alarm cues^50^, as well as morphological responses to elevated predation risk in other taxa^30,53–55^. Deeper bodies in fish increase handling times for predators^56^. For example, the pike *Esox lucius* L., which is also a common predator in minnow habitats, requires more time to handle deep-bodied crucian carp and if given a choice, prefers shallow-bodied carp^19^. Moreover, deeper bodies^63^ as well as short and deep caudal peduncles^57,58^ are suggested to enhance escape locomotion which prevents capture by predators. As dorsal fins can be used for movements independently of the body during manoeuvring, they can generate a substantial portion of additional force for locomotion and furthermore support turning behaviours^64^, the observed plastically increased dorsal fin bases in alarm cue-exposed juvenile and male minnows are likely linked to enhance escape behaviour that includes swimming bursts and sudden turns. The observed larger eyes in adult alarm cue-exposed males match previous results suggesting a plastically increased eye diameter in male cichlids as a consequence of exposure to conspecific alarm cues^30^ and may lead to increased visual acuity^65^, thereby enhancing predator detection^66^. However, this result contrasts the recent observation that when exposed to both visual and olfactory cues of predators, male perch *Perca fluviatilits* decrease their relative eye size whereas females increase it^67^ and in fish from predatory habitats and their offspring, smaller eye size is usually observed^68^, which may arise from the increased conspicuousness of large-eyed individuals to predators^52^. Predator presence selects against larger eyes particularly in open-water habitats where light reflections from large eyes are easily recognizable to predators^67^. This indicates that the observed response in the present study and in Meuthen *et al*.^30^ may either be a result specific to a lack of visual exposure to predators (fish were exposed to chemical alarm cues) or, alternatively, a difference arising from differences in habitats where the benefit due to an increase in visual acuity may outweigh the cost of being more easily recognizable to predators. The latter theory is also in accordance to our observations that juvenile fish do not display a plastic increase in relative eye size whereas only adult males do. That is because juvenile *P. promelas* form free-swimming shoals inhabiting open water whereas adult male minnows occupy breeding habitats below rocks, wood and other vegetation^36^, where the light reflection from their eyes is likely minimized. Lastly, it may be surprising that we did not find any consequence of treatment on body size as this is a frequently observed pattern in the inducible defences of fish^30^. However, in a review of studies concerning predator-induced morphological defences, Bourdeau and Johannson^69^ suggest that the growth response to predation risk is inconclusive: across different aquatic taxa, increases, no changes as well as decreases in growth are observable. For example, in *Carassius auratus*, a smaller body size was observed as a response to either exposure to predatory pike cues or exposure to conspecific alarm cues^70^. Likewise, even within a species, such as *Carassius carassius*, some studies suggest predator-induced increased growth^71–73^, whereas others did not find any evidence of different growth patterns even despite prolonged exposure to predators^18,50,62,74^; only body depth responses were consistently observable across all studies. This suggests that in contrast to the typical body depth response, the growth response is an inducible defence that appears to not just be based on diet^70,74^ but instead is only expressed under certain conditions that are not yet fully understood.

Interestingly, the development of these defensive morphological changes was so pronounced in male *P. promelas* that treatment effects were also significant in adult individuals when both sexes were pooled (see general effect of treatment in Principal Component 6; Table 1), indicating that the previously observed significant responses of predation risk on body depth across sexes in cyprinids^18,50,62^ and other taxa^53–55^ may likewise have been a by-product of a male-specific response. Even the observed treatment effects in juvenile fish of the present study where sexes cannot be discriminated due to a lack of sexual dimorphism at this developmental stage may ultimately arise from treatment effects occurring primarily in male juvenile fish.

Male minnows were not only larger than females but at the same time also had a distinctly different body shape. Males developed a more pronounced head whereas females developed a stouter body shape comprising mainly of a large belly region. This observation matches previously observed patterns of sexual dimorphism in this species. The large head of male *P. promelas* with attached nuptial tubercles aids them in cleaning breeding surfaces and eggs during their allopaternal care while the larger body is beneficial during brood defence^36^, which is an easily noticeable morphological feature that gave this species the common name “fathead minnow”. In contrast, adult females, being fractional spawners^36^, invest largely into egg production, generating up to 500 eggs in a single clutch while being able to spawn new clutches every other day (D. Meuthen, personal observation). This is likely to generate the deep bellies that are observable as the main change in body shape between males and females.

Furthermore, the observed sex-specific morphological response at the onset of sexual maturity is in accordance with theories predicting sex-specific plasticity due to differences in selection pressure between sexes as adults^8–12^. Likewise, morphological antipredator defences manifesting only in male *P. promelas*, similar to other fish studies^28–30^, may be the result of the greater predation intensity acting on male animals that leads to female-biased sex ratios in natural animal populations^23–25^. This is because males are frequently the more conspicuous sex due to their larger size, ornamentation and activity patterns^27^. Similarly, adult male *P. promelas* are larger than females, which could cause the observed offset in morphological plasticity as a consequence of sexual size dimorphism^75^, and males are more active than females during the breeding period: Being a fractional spawner with alloparental care, male *P. promelas* aggressively defend clutches containing eggs from multiple females until they hatch whereas aside from the immediate spawning process, adult females remain free-swimming individuals^36^. Hence, during the breeding period, male *P. promelas* are likely more frequently exposed to predators that aim to feed on the defended clutch and may also feed on adult minnows. This might be a consequence of the fact that defending a clutch requires male minnows to be more stationary, which introduces a trade-off between egg survival and adult survival when it comes to a decision to flee from the predator. This conclusion is supported by a study that found more scarring from attacks by predatory snakes and crayfish in breeding condition males compared to sexually mature female *P. promelas*^40^. Hence, it appears that the observed male-specific morphological modifications provide large benefits during the breeding period. However, as activity patterns are only distinctly different during the breeding period that can be short for wild fathead minnow populations inhabiting northern latitudes, it remains an open question whether the increase in conspicuousness for males arising from sexual size dimorphism alone increases predation risk to an extent that the maintenance of the observed defences is selected for even outside of the breeding period. In this case, it needs to be determined whether the fitness-related costs of the morphological anti-predator phenotype outside of the breeding period outweigh the maintenance and production costs of developing a temporary morphological phenotype^5^. However, as we measured morphology only at 180 days age, which is the onset of sexual maturity in our population, we cannot draw any conclusions about whether the observed morphological modifications in *P. promelas* are permanent or temporary and regress outside the breeding period. Given the theoretical patterns from Fischer *et al*.^7^ who propose that plastic modifications should only be present at the developmental stages where predation pressure is highest, which has been confirmed empirically for fish morphology by Meuthen *et al*.^30^, we suspect that the plastic modifications regress outside of the breeding period in *P. promelas* as well. This may be possible because under perceived predation risk fathead minnows adjust their behaviour (ranging from different shoaling behaviour to altered activity patterns) independent of sex and developmental stage^34,37–44,49,60^, which may be sufficient to compensate for the lower levels of predation risk experienced by both males outside of the breeding period and females while the higher maintenance and production costs of morphological defences^5^ pay off only under high predation pressure. Moreover, that the perceived level of predation pressure was high at the onset of sexual maturity in our experimental fish (i.e. at 5–6 months age) may be a by-product of us having used lab-reared fathead minnows that are able to breed throughout the entire year and where the timing of sexual maturity co-occurs with the onset of breeding. Wild minnows, especially the populations inhabiting aquatic habitats at northern latitudes, typically breed only once a year, during the spring (i.e. at 11–12 months age). Hence, in such wild populations, we would expect the observed sex-specific morphological response to occur at the start of the breeding period in the spring rather than at the onset of sexual maturity during the winter.

Despite the publication of hundreds of studies showing morphological predator-induced plastic changes in phenotypes, our results add to a very small, but growing number of studies that demonstrate this plasticity is specific to particular developmental stages and sex. In fact, similar to another study^30^, we were unable to detect morphological plasticity in female individuals, which suggests that there are a tremendous number of outstanding issues when it comes to understanding phenotypic plasticity. Ignoring sex across morphological plasticity studies may have led to underrated degrees of plasticity present in nature as one sex showing no plasticity may mask plastic responses across whole populations. Hence, future studies, especially those on morphological plasticity, should abandon the widespread approach to pool results across sexes, and focus on revealing whether it is common for males but not females to show plastic responses to environmental change. This may allow us to also figure out potential differences between semelparous and iteroparous species and conclude what factors constrain the ability of female individuals to display a morphological response. We furthermore are in desperate need of additional studies to replicate these results across different environments and taxa so as to allow us a more comprehensive view on general patterns of variation in morphological plasticity that is required to interpret the evolutionary significance of phenotypic plasticity^2^. In this context, the most exciting work will be to determine whether similar patterns of variation in morphological plasticity can be observed when information about the state of the environment is transmitted across generations.

## Supplementary information

Supplementary Information

Supplementary Information
