# Supplementary material for: Predation risk induces age- and sex-specific morphological plastic responses in the fathead minnow Pimephales promelas
Source: Sci Rep. 2019 Oct 25;9:15378. doi: 10.1038/s41598-019-51591-1 (PMC6814781; doi:10.1038/s41598-019-51591-1)
Supplement: Supplementary file 1 — Supplementary Information [file 41598_2019_51591_MOESM1_ESM.docx]

**Supplementary Methods and Results**

**1. Breeding, rearing and alarm cue exposure protocol**

Breeding protocol

In September 2017, we produced fish for our experiments by breeding adult minnows from a stock population housed in the Aquatic Toxicology Research Facility (ATRF), University of Saskatchewan, Saskatoon, Canada. This population was originally obtained from a commercial supplier (Aquatic Research Organisms Inc., Hampton, USA), who purchased them from the United States Environmental Protection Agency, Ohio in 1985, and bred them in captivity for over 34 years, while diversifying them genetically in 1989, 1993, 1999, 2005 and 2017 using fish from different commercial fish hatcheries and research laboratories in Arkansas, Colorado, New York, and Virginia. Laboratory populations such as these are easier to breed as opposed to wild-caught individuals and are unlikely to have a lower propensity for plasticity as exemplified in another cyprinid, the goldfish (*Carassius auratus*). Despite being subjected to selective breeding for over a millennium, goldfish still retain great phenotypic plasticity, likely due to strong selection by predation risk on prey morphology and behaviour^1,2^. We randomly formed pairs of sexually mature fathead minnows and bred them in 26 x 41 x 20 cm tanks filled with approximately 21 l of water. Each tank contained an airstone for oxygen supply and two halves of PVC pipes to be used as breeding caves. Water temperature was kept constant at 25 ± 0.5°C, tanks were illuminated in a 16:8 light:dark cycle (from 6 am to 10 pm) with 800-1000 lux. Every day, sixty percent of water was replaced with dechlorinated tap water. Two to three times per day, fish were fed with frozen bloodworms (Bio-Pure Blood Worms, Hikari Sales Inc., Haywards, CA, USA) until satiety. Breeding caves were checked daily for a period of two weeks and eggs were removed immediately following deposition (see below). In total, we collected 10 clutches from 10 different pairs for our experiment.

Rearing and documentation protocol

Eggs were removed from the breeding caves by gently rubbing them off with a moist rubber glove. Afterwards, we split them into two equally sized groups with 58-140 eggs each and incubated them in a plastic cup (diameter 12 cm, height 8.5 cm) together with 500 ml of water and a gently bubbling airstone. Every day, we replaced 80% of the water with fresh dechlorinated tap water. After hatching, fish were exposed to two different predation risk treatments (see below). By using a split-clutch design, we were able to control for genetic variation during our study of inducible morphological defences. Following hatching, we generated two replicates per treatment containing 10 fry each and transferred them into new cups; in total we used 40 fry per clutch and family for our experiment. All additional fry were used for a third replicate that was density-unmatched (24-118 fry) and raised in 20 x 30 x 12.5 cm (L x W x H) tanks (5 l water volume) but otherwise treated the same as the first two replicates. In the case of mortality during the first 39 days after hatching, we replaced the dead fish with a random individual from the third replicate; fish mortality in this period was similar across treatments (median, IQR; alarm cue-exposed fish: 12.5 %, IQR 5 – 33.8 %; control fish: 7.5 %, IQR 5 – 25 %; paired Wilcoxon test: V = 11.500, p = 0.343). At 39 days age, groups of fish were transferred from their cups to 34.5 x 27.0 x 18.5 cm (L x W x H) tanks (PC90 10l with LID90I-4 blue poly lid, Pentair Aquatic Eco-Systems, Atlanta, USA) containing 375 ml of gravel (mean±SD weight: 540 ± 15 g), an airstone and two breeding tiles (halved PVC pipe pieces, diameter 9.5 cm, length 7.5 cm) so as to conform to the increased space requirements of growing fish. Here, they received a 25 % water change with fresh dechlorinated tap water weekly. Fish were generally fed *ad libitum* so as to avoid nutritional limits on the formation of morphological defenses^2^; hence the amount of food was sequentially increased over development: 1-3d: 1µl sieved, pure *Artemia* nauplii/fish; 4-18d: 10µl sieved *Artemia* nauplii /fish; 18-39d: 20 µl sieved *Artemia* nauplii /fish; 39 d onwards: *ad libitum* commercial flake food (Nutrafin A6840 Max Goldfish Flakes, Hagen, Mansfield, USA). Throughout rearing, fish in different tanks did not have visual or olfactory contact.

Perceived predation risk treatment

Treatments consisted of two different chemical stimuli to which fish were exposed five days a week: either alarm cues (AC) or a distilled water control (DW). The purpose of the distilled water treatment was to control for potentially confounding effects induced by the water disturbance that occurs during the introduction of alarm cues into tanks. Alarm cues were produced from four adult wild-caught *P. promelas* (mean ± SD total length: 55.9 ± 6.4 mm, weight: 1.83 ± 0.69 g) that were caught with Gee's inverted minnow traps in August 2017 at Feedlot Pond (52°09'23.4"N, 106°37'04.5"W) on the University of Saskatchewan campus. Minnows from this population produce alarm cues that induce antipredator responses in conspecifics^3-5^. Donor minnows were kept for one month at the RJF Centre for Aquatic Ecology in 1700-L pools (diameter: 187 cm) at 20 ± 1 °C. Here, they were fed with the same commercial flake food as before; water was exchanged continuously (thereby, 30% of water was replaced per day). Afterwards, we picked four random individuals as skin donors to avoid potential individual or sex effects. We euthanized donors with a blow to the head followed by cervical dislocation. Skin fillets were generated by making small incisions with a scalpel and pulling off skin with tweezers. The area of these fillets was then measured by placing them in a Petri dish containing graph paper as a size standard, spreading them out and photographing them with a digital camera (Olympus E-3 with Zuiko Digital ED 50mm 1:2.0 macro objective). We then used ImageJ software (Rasband 1997-2014, National Institutes of Health, Maryland, USA) on these photographs to obtain measures of skin area as it converts digital measurements to metric units according to size standards. Following standard alarm cue production procedures across fish taxa^6-11^, we then ground skin fillets (total skin area: 8.57 cm², skin weight: 0.128 g) together with some distilled water using a homogenizer (Polytron PT2500 E, Kinematica, Luzern, Switzerland). The homogenate was then passed through filter floss to remove solid particles and further diluted with distilled water to generate a total volume of 5088 ml. This dilution equals a concentration of 0.0017 cm² skin/ml. Aliquots containing 1 ml of this diluted extract were then frozen at -20°C until use, a temperature that retains the ability of alarm cues to induce an antipredator response^12^. Similarly, we produced 1 ml aliquots of distilled water for the water control. On the day of use, these aliquots were thawed, mixed with 254 ml dechlorinated tap water and added to the experimental tanks in a volume proportional to tank size (0.250 ml for 500 ml cups, 2.5 ml for 5 l tanks and 5 ml for 10 l tanks) so as to generate a concentration of 3.302 x 10^-6^ cm² skin / l within each tank. This concentration was found to elicit recognizable behavioural antipredator responses in *P. promelas*^4,5^.

**2. Photographic documentation**

Fish were first photographed at 18 days age because *P. promelas* complete their larval development and enter the juvenile phase at 443 hours post-hatch^13^. For this purpose, individual fish were transferred into water-filled distortion-free and orthochromatic quartz glass cuvettes (100-OS, outer dimensions 1.25x0.75x4.4 cm, inner dimensions 0.95x0.5x4.4 cm, Hellma, Germany). Cuvettes with fish were then placed on an L-shaped piece of black cardboard. Standardized illumination was then provided by a 13W LED lamp (S9434, Long Neck Flood 40°, 5000 K, Satco, USA) from 3 cm above the cuvette (light incidence in a 90° angle with the fish). Cuvette size was used as a size standard. Multiple pictures per individual were then taken with a digital camera (Olympus E-3 with Zuiko Digital ED 50mm 1:2.0 macro objective) in ORF (Olympus RAW) format. All juvenile fish were always obtained from a single replicate throughout.

Fish were photographed a second time as adults at 180 days age, at which we observed the first individuals starting to develop the morphological changes indicating sexual maturity^14^. Here, pictures of adult fish were taken inside of a 34.5 x 27.0 x 18.5 cm tank under standardized illumination provided by the same lamps as mentioned before positioned in a distance of 15 cm (light incidence in a 45° angle with the fish). Fish were held into place within an 87 x 73 x 10 mm central area using a perforated plastic pane with attached sponge stripes. White and size standards were placed inside the water close to the fish. Pictures were taken as described above. Due to juvenile mortality and sex effects, we always photographed fish from both replicates to obtain adult pictures of a sufficiently large sample size for both sexes.

For analysis, we selected the best photograph for each individual, where it was perfectly perpendicular to the camera and where fins were fully spread out. In total, 10 fish per treatment from 10 different families were raised per replicate; we photographed one full replicate for juvenile fish and thus analyzed 200 photographs (100 per treatment) of juveniles. For adults, we photographed all 378 available fish but to be able to consider sex effects in our analysis, we analyzed only photographs of those fish whose sex was clearly recognizable by their external morphology (as selected by an observer naïve to the treatment). Thus, 267 photographs of adult fish could be analyzed (alarm-cue exposed males n = 85, control males n = 72, alarm cue-exposed females n = 53, control females n = 57).

**3. Analysis of photographs**

Photographs were imported into Stepok RAW Importer (Stepok Image Lab, New Zealand) and converted in PNG format. Afterwards, using tpsUtil 1.74 (F. James Rohlf, Stony Brook University, New York, USA) we built a tps file from images. Then, in tpsDig2 2.30 (F. James Rohlf, Stony Brook University, New York, USA) we first calibrated the image according to the size standards and then placed thirteen landmarks (see Fig. 1) for geometric morphometry analysis. Preliminary analyses revealed that incorporating ontogenetic changes into the shape analysis (i.e. also analyzing deformation in body shape across ontogeny) leads to biphasic, non-normally distributed data and the amount of variables to control for (repeated-measure design with age × treatment × sex interactions and tank random effects) was too large relative to the available sample size, hence preventing any models to converge. Furthermore, the aim of our study was not to confirm the already well-known ontogenetic shifts in body shape between larval and adult fish; hence, we analyzed juvenile and adult body shape separately. Accordingly, data from juvenile and adult fish was then separately imported in CoordGen 8 (Integrated Morphometrics Package Suite, H. David Sheets, Canisius College, New York, USA) to conduct a generalized Procrustes superimposition on the landmarks. This superimposition removes differences in location, size and orientation between individual landmark combinations by translating, scaling and rotating the landmark data^15,16^. The Procrustes data was then imported in CVAGen 8 (Integrated Morphometrics Package Suite, H. David Sheets, Canisius College, New York, USA) to conduct a Canonical Variate analysis (CVA) and extract the canonical variates (CV) for analysis. CVA is a generalization of a Linear Discriminant analysis that maximizes the proportion of between-group sum of squares to within-group sum of squares. Thereby, this multivariate analysis of variance maximizes the variation among pre-determined groups and allows visualizing shape changes between these groups and testing whether the data supports the assignment to the same groups^15^. During this analysis, we assigned individual shapes a priori to the following groups: alarm cue-exposed juveniles (n = 100), control juveniles (n = 100), alarm-cue exposed males (n = 85), control males (n = 72), alarm cue-exposed females (n = 53) and control females (n = 57). By using such large sample sizes that outweigh the number of landmarks (13, see Fig. 1), we have ensured that the assessment of group separation is reliable^17^. For visualization and testing, we only used significant CVs, which was one CV for the juvenile dataset and three CVs for the adult dataset. As only similarities between significant CVs and principal components (PC) from a principal component analysis (PCA) confirm that the axes of variation among individuals relate to axes of statistically significant differences in body shape^15^ and thereby ensure a biologically relevant interpretation of variation in body shape, we also conducted a PCA. For this purpose, we also imported the Procrustes data into PCAGen 8 (Integrated Morphometrics Package Suite, H. David Sheets, Canisius College, New York, USA). A PCA is an eigenvalue decomposition of the variance-covariance matrix across individuals. Because the Kaiser criterion^18^ is now considered unsuitable to select significant principal components^19^, we instead visually inspected a Scree plot of the percentage of variance explained by each of the principal components with the rule-of-thumb to use up to the last component before the plot flattens out. Furthermore, we aimed to analyze at least 90% of cumulative variance in shape as well as to not exclude any component with more than 2% explanatory power. Hence, for the juvenile dataset, we retained the first eleven PCs for analysis, together explaining 92.73 % of total variance with the eleventh PC by itself explaining 2.02 % of variation in body shape. Likewise, for the adult dataset, we retained the first eleven PCs for analysis; together they accounted for 90.87% of the observed morphological variation and the eleventh PC by itself explained 2.05% of variation in body shape. Additionally, body size was approximated by using centroid size, which is the square root of the sum of the squared distances between each landmark and the centroid (i.e. the centre between all landmarks). The benefit of using centroid size as a size measure is that it is mathematically uncorrelated with shape as opposed to traditional measures of body size^15^.

**Figure S1.** Morphological differentiation along the first canonical variate axis of 18-day-old juvenile *Pimephales promelas* subject to different levels of perceived predation risk: distilled water (n=100; white bar) and conspecific alarm cues (n=100; dashed bar). Mean values ± SE as well as depictions of the deformation in body shape along the axis are shown. Schematic fins and eyes (gray) are included in these figures to better visualize measured changes in fin position and fin base width. The asterisks indicate p ˂ 0.001.


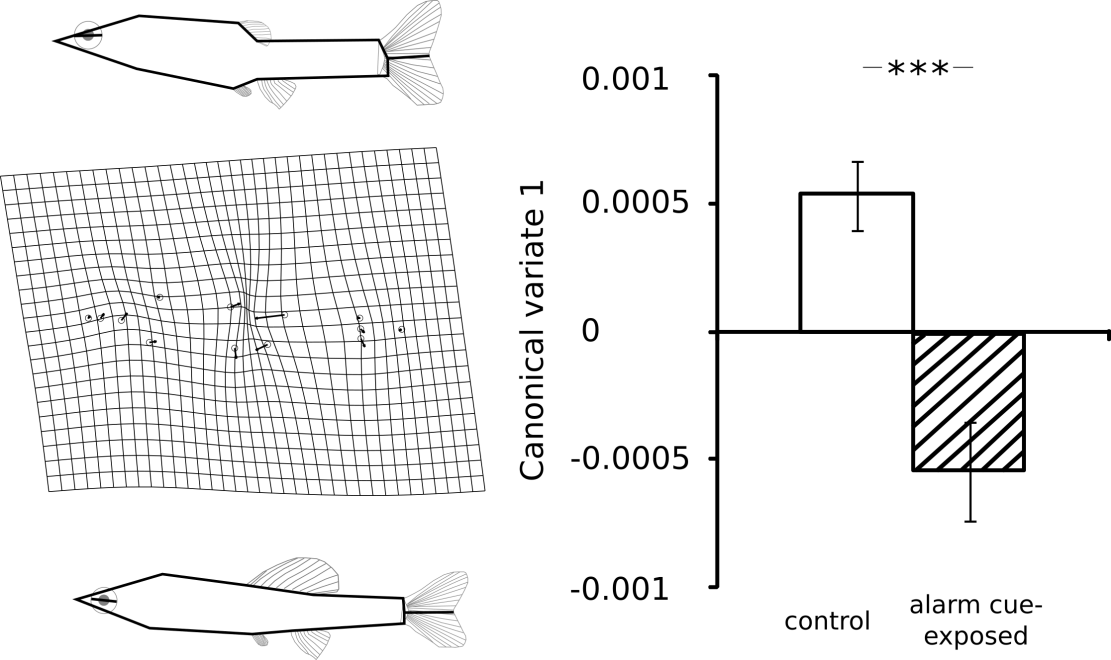


**Figure S2.** Morphological differentiation along the first two canonical variate (CV) axes of male and female 180-day-old adult *Pimephales promelas* subject to different levels of perceived predation risk: distilled water (male n=72, gray crosses; female n=57, gray stars) and conspecific alarm cues (male n=86, gray circles; female n=53, gray rectangles). Black symbols indicate mean values; standard errors are smaller than symbol size and thus not depicted. Shown are also deformation grids with displacement vectors for CV1 (a) and CV2 (b) as well as depictions of the deformation in body shape along both axes. Schematic fins and eyes (gray) are included in these figures to better visualize measured changes in fin position and fin base width.


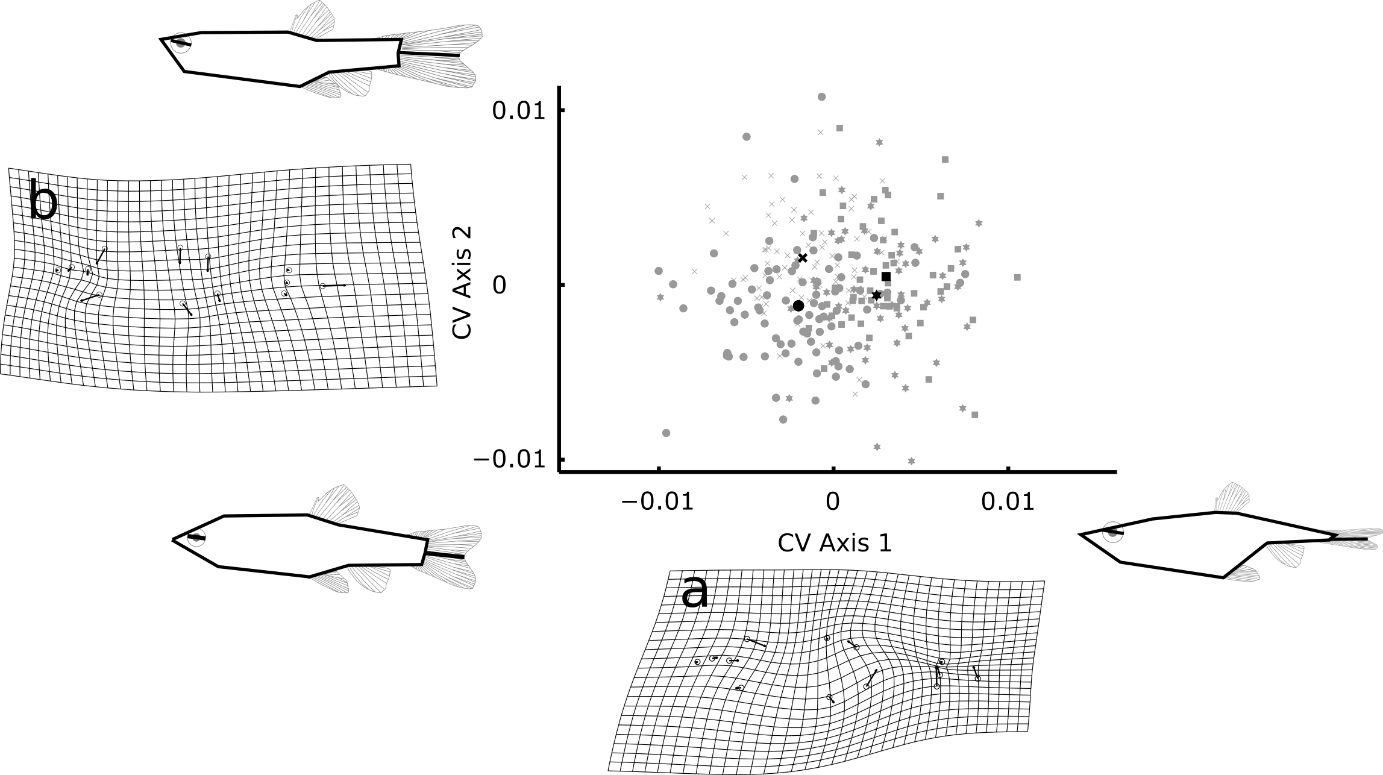


**Figure S3.** Morphological differentiation along the first and the third canonical variate (CV) axes of male and female 180-day-old adult *Pimephales promelas* subject to different levels of perceived predation risk: distilled water (male n=72, gray crosses; female n=57, gray stars) and conspecific alarm cues (male n=86, gray circles; female n=53, gray rectangles). Black symbols indicate mean values; standard errors are smaller than symbol size and thus not depicted. Shown are also deformation grids with displacement vectors for CV1 (a) and CV2 (b) as well as depictions of the deformation in body shape along both axes. Schematic fins and eyes (gray) are included in these figures to better visualize measured changes in fin position and fin base width.


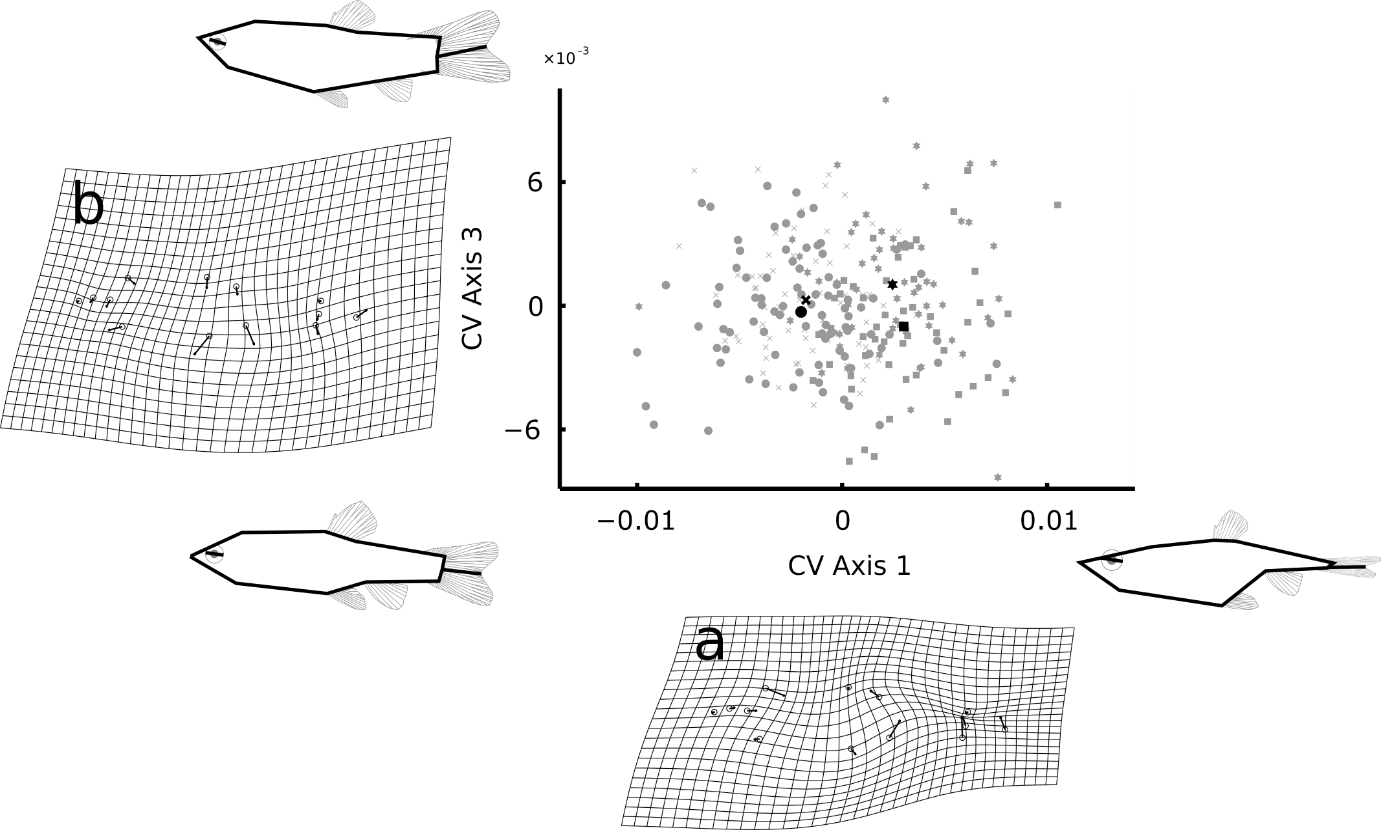


**References**

1. Chivers, D. P., Zhao, X. & Ferrari, M. C. O. Linking morphological and behavioural defences: Prey fish detect the morphology of conspecifics in the odour signature of their predators. *Ethology* **113**, 733-739 (2007).

2. Chivers, D. P., Zhao, X., Brown, G. E., Marchant, T. A. & Ferrari, M. C. O. Predator-induced changes in morphology of a prey fish: the effects of food level and temporal frequency of predation risk. *Evol. Ecol.* **22**, 561-574 (2008).

3. Chivers, D. P. & Smith, R. J. F. The role of experience and chemical alarm signaling in predator recognition by fathead minnows, *Pimephales promelas*. *J. Fish Biol.* **44**, 273-285 (1994).

4. Ferrari, M. C. O., Trowell, J. J., Brown, G. E. & Chivers, D. P. The role of learning in the development of threat-sensitive predator avoidance by fathead minnows. *Anim. Behav.* **70**, 777-784 (2005).

5. Meuthen, D., Ferrari, M. C. O., Lane, T. & Chivers, D. P. Plasticity of boldness: high perceived risk eliminates a relationship between boldness and body size in fathead minnows. *Anim. Behav.* **147**, 25-32 (2019).

6. Manek, A. K., Ferrari, M. C. O., Sereda, J. M., Niyogi, S. & Chivers, D. P. The effects of ultraviolet radiation on a freshwater prey fish: physiological stress response, club cell investment, and alarm cue production. *Biol. J. Linn. Soc.* **105**, 832-841 (2012).

7. O'Connor, C. M., Reddon, A. R., Odetunde, A., Jindal, S. & Balshine, S. Social cichlid fish change behaviour in response to a visual predator stimulus, but not the odour of damaged conspecifics. *Behav. Process.* **121**, 21-29 (2015).

8. Barreto, R. E., Barbosa, A., Giassi, A. C. C. & Hoffmann, A. The 'club' cell and behavioural and physiological responses to chemical alarm cues in the Nile tilapia. *Mar. Freshw. Behav. Physiol.* **43**, 75-81 (2010).

9. Crane, A. L. & Ferrari, M. C. O. Minnows trust conspecifics more than themselves when faced with conflicting information about predation risk. *Anim. Behav.* **100**, 184-190 (2015).

10. Stephenson, J. F. Keeping eyes peeled: guppies exposed to chemical alarm cue are more responsive to ambiguous visual cues. *Behav. Ecol. Sociobiol.* **70**, 575-584 (2016).

11. Brown, G. E., Demers, E. E., Joyce, B. J., Ferrari, M. C. O. & Chivers, D. P. Retention of neophobic predator recognition in juvenile convict cichlids: effects of background risk and recent experience. *Anim. Cogn.* **18**, 1331-1338 (2015).

12. Lawrence, B. J. & Smith, R. J. F. Behavioral-response of solitary fathead minnows, *Pimephales promelas*, to alarm substance. *J. Chem. Ecol.* **15**, 209-219 (1989).

13. Devlin, E. W., Brammer, J. D., Puyear, R. L. & McKim, J. M. Prehatching development of the fathead minnow *Pimephales promelas* Rafinesque. *EPA/600/R-96/079. United States Environmental Protection Agency, Washington, USA* (1996).

14. Scott, W. B. & Crossman, E. J. *Freshwater fishes of Canada*. (Galt House Publications Ltd, 1998).

15. Webster, M. & Sheets, H. D. A practical introduction to landmark-based geometric morphometrics. *Paleontol. Soc. Pap.* **16**, 163-188 (2010).

16. Zelditch, M. L., Swiderski, D. L., Sheets, H. D. & Fink, W. L. *Geometric morphometrics for biologists*. (Academic Press, 2004).

17. Mitteroecker, P. & Bookstein, F. Linear discrimination, ordination, and the visualization of selection gradients in modern morphometrics. *Evol. Biol.* **38**, 100-114 (2011).

18. Kaiser, H. F. The application of electronic computers to factor analysis. *Educ. Psychol. Meas.* **20**, 141-151 (1960).

19. Morton, F. B. & Altschul, D. Data reduction analyses of animal behaviour: avoiding Kaiser's criterion and adopting more robust automated methods. *Anim. Behav.* **149**, 89-95 (2019).
